# Examining the role of emotional intelligence as a moderator for virtual communication and decision making effectiveness during the COVID-19 crisis: revisiting task technology fit theory

**DOI:** 10.1007/s10479-021-04216-8

**Published:** 2021-09-13

**Authors:** Uma Warrier, Anand Shankar, H. M. Belal

**Affiliations:** 1 CMS Business School, Faculty of Management Studies, JAIN (Deemed to be) University, Bangalore, India; 2 TATA Group, Pune, Maharashtra India; 3 Liverpool Business School, Liverpool John Moores University, Liverpool, UK

**Keywords:** Emotional intelligence, Virtual communication, Task technology fit theory, Hierarchical regression, COVID-19, Pandemic, Disaster

## Abstract

The COVID 19 has brought unprecedented changes in the way we communicate. There is a greater accent on Virtual communication. This paper aims to establish a relationship between Emotional intelligence and the effectiveness of Virtual communication on Decision making. This empirical study is based on a sample drawn from 296 working professionals at five different levels of organizational hierarchy. A standardized questionnaire (ɑ = 0.824) was used to collect the responses of Emotional intelligence, Virtual communication, and Decision-making effectiveness. Hierarchical regression using PROCESS Macro model 1 was used to identify the moderating effect of Emotional intelligence on Virtual communication and Decision making effectiveness. Since the *p*-value (*p* ≤ .007) is found significant, Emotional intelligence acts as a moderator that affects the strength of the relationship between Virtual communication effectiveness and Decision making. Validation of Task Technology fit theory is the theoretical implication of the study. Manipulation of individual dimensions in the model can reduce the dependence on technology for task completion with enhanced performance effectiveness. The findings are relevant to educators, consultants, and any professional who need to adapt Virtual communication platforms on an ongoing basis. Since work-life balance is projected as a constraint in this study, policymakers can consider policy amendments to reduce the stress caused due to Virtual communication which intrudes into their personal space. This empirical study is the first of its kind to benchmark the organizational practice of Emotional intelligence training to enhance Virtual communication and Decision making effectiveness during unprecedented times of pandemic.

## Introduction

The world is reeling under an unprecedented crisis due to COVID 19 pandemic and the effects are forecasted to be long lasting (Dubey et al., 2021a, 2021b; Queiroz et al., 2020; Sparrow, 2020). The human community is grappling with multitudes of challenges posed both on the personal as well as in the occupational front. Business continuity emerges as the biggest challenge for business houses during pandemic (Sikich, 2008). Social and economic uncertainties due to the pandemic has had an impact on societies and business (Ivanov, 2021; Martin et al., 2020) and in the same way, it has disrupted critical social systems or the infrastructure and environment (Dubey et al., 2019; Gunasekaran et al., 2018). The government initiated lockdown as well as social distancing, necessitated organizations to facilitate their employees to work effectively from the safety of their homes. Work from home is not a new practice in many organizations as technology advancements have provided remote access to office work set-up. Besides, industry 4.0 has adopted computers and automation and enhanced its growth of practice with smart and autonomous systems fuelled by data and machine learning (Wollschlaeger, 2017). Management of virtual team members necessitates the use of a different set of protocols and practices for technology adoption, building trust and team motivation for greater effectiveness (Rezgui, 2007). The importance of Emotional intelligence (EI) on effective Virtual communication (VC) is found in the recent research literature (Alward & Phelps, 2019; Barbara, 2018; Cole et al., 2015). This paper aims to establish a relationship between ‘EI and the effectiveness of VC in Decision making (DM)’.

Artificial intelligence tools have increased the incidence of working away from the physical infrastructure of the office (Sipior, 2020) and it helps as a decision support system (Gupta et al., 2021). COVID 19 initiated a lockdown fast-tracked remote working pattern with the majority of the workforce left with no choice but to work from virtual settings. This unexpected shift from working in physical teams to virtual teams and organizational adaption (Carley, 1997) can be unsettling. Coupled with this, there are no reference materials or operating guidelines available to effectively communicate in an exclusive VC environment. There are no policies and procedures available to benchmark and amend. The transition from working at an office workstation to home and maintaining the same level of productivity, communication, mutual trust can be challenging. It requires a lot more planning and coordination in comparison to face-to-face meetings. The motivational level of employees to adopt technology-based communication would vary, thereby time taken to adapt new technology for seamless communication would also vary in the organization. Despite these challenges, organizations are left with no choice but to resort to work from home (WFH) as the new normal and not as an exception due to the social distancing stipulations necessitated by the pandemic (Akala, 2020). Using advanced-level IT tools and/or digital innovation through artificial intelligence, blockchain, cloud, and data analytics (Akter et al., 2020) may enhance resilience and reduce disruption risks (Katsaliaki et al., 2021) by ensuring the design and delivery of emergency services (Fosso Wamba et al., 2019) in cases of any natural calamity. Companies like TCS have communicated their plan to have 75% of the employees work from home by the year 2025, which puts heavy dependence on VC (Khetarpal, 2020). The educational sector had to adapt synchronous modes of teaching by using video conferencing platforms like Webex, Zoom, Google meet, and a host of other platforms. Asynchronous learning platforms/providers like Coursera have also shown greater adoption (Shah, 2020). Changes in the modes of communications are sudden and unprecedented, thereby causing a lot of stress to cope with the mechanisms of adapting to this change. Warrier et al. (2021) assert that augmenting EI competencies will help in enhanced resilience to deal with adversities. There are reports of technostress due to intense scrutiny and extended hours of work during the lockdown (Kalia, 2020). Similar results were found in earlier studies where digital technology increased productivity at the same time increased technostress (Tarafdar, 2007). Technostress happens when employees are expected to quickly adapt to newer technologies. In this, they need to be accessible for extended periods and are expected to do effective multitasking. Virtual teams are understood to have four major areas of challenges: Project management, Technology, Communication, and Culture (Leidner & Kayworth, 2000). This study has a greater accent on human factors than technology connected factors that pose challenges to the effectiveness of VC and effective DM.

Virtual meetings are understood as meetings where ‘participants may or may not be a part of a permanent team and who interact with each other non-simultaneously using shared data’ (Dubey et al., 2021a, 2021b; Munro & Swartzman, 2013). Since the lockdown, organizations rely heavily on VC in the form of video conferencing tools, chat messages on smartphones, chat apps, emails, and telecalls. VC in the context of this study is a combination of quality of communication, speed and reach of communication, the convenience of communication, and ultimately meeting the purpose of communication. As the global economy gets more dependent on the functionality, utility, and effectiveness of these VC media in organizational DM, there is a huge scope for primary research to be conducted to understand the effectiveness of VC. It is also becoming imperative to understand the moderating human factors that can augment the effectiveness of VC and DM. EI is one such factor that impacts the effectiveness of VC. EI is ranked in the top ten employability skills by the world economic forum for the past few years. There is a large body of research that confirms the beneficial effects of EI in workplace settings. EI is found to advantageous for entrepreneurial success also (Karia, 2021). In a qualitative phenomenological study to explore the effectiveness of virtual teams, major themes emerged were EI, virtual leadership competencies, and communication (Alward & Phelps, 2019). A major body of research in VC has been conducted in the E-learning context, like benefits of E-learning over time tested conventional learning mode (Xu & Jaggars, 2016; Zhang & Nunamaker, 2003; Lu et al., 2003).

Conversely, many research studies find E-learning disadvantageous (Atchley et al., 2013; Xu & Jaggars, 2016). Further, no significant difference in E-learning usage or conventional learning was found in another current study (Paul & Jefferson, 2019). This indicates a higher concentration of research in the learning context of VC. Besides, Schiller and Mandviwalla (2007) recommend that future research on virtual teams be theory-based, as much of the published research is not grounded on a theory base. Since there is a paucity of research in VC and DM among working professionals to increase efficiency and performance by checking the moderation effect of Emotional intelligence, authors identified population void, theoretical void, and knowledge void (Miles, 2017). Perception of team members on effective DM in virtual context is a research lacuna (Alge et al., 2003). Good relational development of a team is vital for a team's success, using computer-mediated communication (Guo et al., 2009).

Therefore, this study is an attempt to understand *‘how EI moderates the relationship between VC effectiveness and DM effectiveness’* among working professionals who use VC as the only form of communication for task completion. Published research spanning over two decades were studied to fulfil the objectives of the research. Applying task technology fit (TTF) theory, a conceptual framework is developed for enhancing VC effectiveness on DM by improving EI. A research question, *“Does Emotional Intelligence moderate the relationship between virtual communication and decision making effectiveness?”* is developed for this study. Two hypotheses developed for the study are H1: Virtual communication effectiveness influences Decision making effectiveness (DME) and H2: EI moderates the relationship between VCE and DME.

The following section presents a theoretical framework and literature review on EI, virtual and face to face communication, and DM effectiveness followed by developing the research question. In the next section, the description of the research methodology used to collect data and analysis is presented. Post this section, the analysis of results are shown followed by a discussion, implications, conclusion, limitations, and scope for future research in the subsequent sections.

## Theoretical model and hypothesises development

Theoretical grounding is important for a research study, as it lays a foundation for a conceptual framework for deriving hypotheses. This study draws insights from TTF theory and an EI model. A scoping review study confirms the high potential of application of TTF in varied environmental contexts in both industry and academia (Spies et al., 2020).

TTF (Goodhue et al., 2000) deals with the connectedness between requirements of a specific task and the functionalities of technology chosen to complete the task as well as individual characteristics of the employee who undertakes the task. The functionality of technology refers to functional capability, specifically enabled by the chosen technology. Individual characteristics refer to the dimensions like socio-economic, attitudinal and motivational aspects of the employee. The theory explains how and to what extent the technology supports an individual to complete the task. The theory also explains an interplay of the task, technology and individual in the performance (Goodhue et al., 2000). TTF maps the variables of interest of the current study. It is used in the context of performance prediction in technological settings. This study attempts to identify whether greater levels of individual characteristics can bring about improved Technology-task fit and thereby greater performance.

The EI model of Bar-On (2006) is used as the theoretical framework for the study. Bar-On identifies EI as a combination of emotional and social skills that involve understanding and expression of self and others, interpersonal interaction, ability to adapt to change and deal with stress, and staying happy and optimistic in the face of adversities.

Bar-On’s EI model deals with emotional and social abilities that help in self-awareness and social awareness. It focuses on the ability to deal with strong emotions, adaptability, and change management skills and problem-solving ability of personal or social nature. Bar-On's EI model has five meta factors that can be further divided into 15 skills, competencies, and facilitators which are closely related. The intrapersonal meta factor consists of Self-regard, Self-awareness, Assertiveness, Independence, and Self-actualization. The interpersonal dimension consists of Empathy, Social responsibility, and Interpersonal relationship. The third meta factor Stress management consists of Stress tolerance and Impulse control. The meta factor Adaptability consists of Reality testing, Flexibility, and Problem-solving. The final meta factor General mood deals with Optimism and Happiness.

VC is here to stay, whether one likes it or not. Dennis et al. (2008) assert that VC is a better source for generating ideas, options, and alternatives. VC using video conferencing with shared mental models is found to be as effective as Face to Face (F2F) communication in teams in China, where the members who communicate see each other while communicating (Guo et al., 2009).

### Virtual communication versus Face-to-Face communication (F2F)

Communicating in a virtual platform is not devoid of complications in communicating with each other. It may lead to performance breakdown if not handled effectively. It is bound to cause psychological distancing, apart from the concerns of space and time zone that pose coordination difficulties. The organizational members need to upgrade the technology skill sets to adapt to the technology used in virtual team meetings. According to Acai et al. (2018), the integrity of DM using VC modes like video conferencing or audio calls is uncertain. Again, Lepsinger and DeRosa (2010) found that group members tend to exhibit Social loafing—A phenomena where the individual effort exerted by the team member is reduced intentionally while working as a team, especially in the VC context. An important challenge of VC is lack of social cues and was researched by De Jong et al. (2016). Research reveals that building mutual trust can pose a big problem among virtual team members, as they are deprived of the social cues in virtual settings. Hambley et al. (2007) report in their study that the use of richer communication media did not bring out greater task performance when the results of F2F and VC were compared. When it comes to return on investment (ROI), VC seems to be the preferred mode of communication, especially in the consulting field. Research studies indicate that projects that use virtual consulting fetched significantly additional revenue than conventional consulting projects (Boh et al., 2007). VC is found to be less effective in bringing demonstrably correct solutions in comparison to F2F communication, and F2F communication is found to be more effective in all DM behaviors (O’Neill, 2016). Most of the literature review studies on VC primarily focus on technology use, however, it is found that the physical factors of distance communication are closely linked to social, cognitive, and emotional challenges.

### Emotional intelligence in virtual communication

An increase in the magnitude of the virtual workforce necessitates exploration of dimensions that augment the effectiveness of VC. A qualitative phenomenological study identifies EI as one of the major themes on VC effectiveness. (Alward & Phelps, 2019). Blended learning that heavily depends on virtual teaching/learning makes use of the EI of tutors. Tutors with high scores of EI were found to be helpful in effective tutoring in blended learning. Many similar results indicate that social competence, which is an EI dimension is highly desirable in the online teaching and learning process. (Bawane & Spector, 2009; Berge, 2009; Guasch et al., 2010; Schichtel, 2010; Varvel, 2007; Youde, 2016). The emergence of EI as a key to successful VC was implied in a research study by London (2012). A study conducted in Thailand (Sunindijo et al., 2007) revealed that high EI in team members helps them to use communication more effectively and thereby get the best performance out of the team.

### Emotional intelligence in decision making

Emotions and cognition are found to be closely linked (Frasson & Chalfoun, 2010) and this underlines the need for studying EI in the context of DM. The role of emotions in DM is an interesting context of research. Emotions improve the relationship between objects and ideas, thereby improved rigor and efficiency in DM (Isen, 2000). Positive effects are also found to create a highly flexible and divergent thought process, which in turn improves the quality of DM (Pekrun, 2005). A model for building intelligent emotional awareness for improved virtual learning is proposed by Daradoumis (2013). The ability to regulate emotions is found to be a prime indicator of efficiency in DM (Gross & Thompson, 2007; Spicer & Sadler-Smith, 2005). Emotional regulation is also found to impact decisions on financial investments. Investors and traders who have better self-regulation have superior DM performance (Seo et al., 2004; Fenton et al., 2011). EI is found to influence intuitive DM (El Othman et al., 2020; Khan et al., 2016). Intuition, which is a dimension of the intrapersonal dimension of EI is found to affect DM (Dane & Pratt, 2007). EI plays a vital role in DM among the working professionals in the health sector (Fatma, 2019). Stress is found to have an impeding effect on DM under uncertainty (Hewitt et al., 2021) by restricting social cue sampling, decreased vigilance, and impairs the capacity of working memory, leading to premature closure of appraisal of options available (Kontogiannis & Kossiavelou, 1999). Similar results were found by Keinan et al (1987), where they found a tendency to offer a solution before evaluating all the available options. Studies show that an individual's mood impacts DM and judgment (Ashby et al., 1999; Forgas, 1995; Wyer et al., 1999) and also argues that interpersonal interaction will influence judgment and DM through different affect infusions.

As evident from the literature review, there is a paucity of research in the field of EI as a moderator between VC and DM effectiveness. Hence the current study aims to fill the knowledge gap in this research area. A research question *“Does EI moderate the relationship between VC and DM effectiveness?”* is developed for this study. Two hypotheses developed for the study are H1: Virtual communication effectiveness (VCE) influences Decision making effectiveness (DME) and H2: EI moderates the relationship between VCE and DME.

## Research design

A cross-sectional study spanning between April to July 2020 was conducted. This empirical paper is based on the responses from participants who truly rely on VC for completing the organizational deliverables. The participants are assigned randomly from four industries: IT, Academics, Health Care, and Financial Services. Working professionals who use VC for day-to-day work transactions are considered the inclusion criteria. Students and other non-working professionals who use VC during the lockdown period were excluded from the study. Email address of the employees of the companies in each sector was used as a sampling frame, and computer-generated random numbers were used to recruit the participants for the study. Participants were informed about the purpose of the study and informed consent was sent before collecting the responses. Participants were assured anonymity, and they were informed that there is “No right or wrong answer” to reduce common method bias (Podsakoff et al., 2003). Participation in this study was voluntary and no incentives were provided to the participants. The self-administered questionnaire in English was sent to 450 respondents electronically and the response rate was 66%. Response rates above 60%, is considered as a “good response rate” and has a lesser non-response bias (Babbie, 2007; Groves, 2006). After data cleaning, 296 responses were considered for data analysis. Working professionals who use VC for the day to day work transactions were considered as the inclusion criteria. Students and other non-working professionals who use VC during the lockdown period were excluded from the study.

Study variables identified are VCE (independent variable), DME (dependent variable), and EI (moderating variable). The questionnaire used for the study assessed socio-demographic details of the participants, apart from the study variables. A set of 42 questions were developed by the researcher to capture dependent, independent and moderating variables. Participants were administered this questionnaire to capture their perspective on the effectiveness of VC (16 items), their opinion of the effectiveness of DM (six items), and another set of questions to measure their level of EI (20 items). EI questions were developed based on Bar-On’s model of EI. The items were on five-point Likert scale, ranging from one (strongly disagree) and five (strongly agree). The use of a five-point rating scale was considered apt for this study as it increases response rate and quality and reduces respondents’ “Frustration level” (Babakus & Mangold, 1992). A pilot study with 50 respondents were conducted for face validity. Post the pilot study, some of the items were reworded based on the suggestions on clarity of questions. Cronbach alpha's reliability was assessed by Cronbach alpha using the split-half reliability method, and the scale was found reliable (Cronbach alpha 0.824). Items were validated by exploratory factor analysis. The estimate values of the items of EI, VC, and DM that are below the threshold limits of 0.5 were dropped. As per Hair et al. (2010), the estimated values of any item/question should be above 0.700. But it can be acceptable in rare cases with above 0.500 (Ertz et al., 2016; Chen & Tsai, 2007). Hence, after the item analysis and exploratory factor analysis, six items were dropped from the EI questionnaire, seven items were dropped from the VC questionnaire, three items were dropped from the DM questionnaire that had an Eigenvalue less than 1 (Girden & Kabacoff, 2010). The constructs are formative, as the causal indicators jointly influence the construct (MacKenzie et al., 2005). The final test instrument consists of 26 questions. Skewness, Kurtosis, and Normality were checked before data analysis, and the sample was normally distributed (Table 1).

**Table 1:** Socio-demographic profile of participants (N = 296)

*Gender*			
Male (1)	163	55.1%	Average age 35.50 years (min. age 17 & max. age 70)
Female (2)	133	44.9%	Average exp.287 months (23.91 years)
*Hierarchy level*			
Team members-Individual contributors (1)	104	35.13%	
Junior management-First-time managers (2)	39	13.17%	
Middle management-Managers of managers (3)	59	19.93%	
Senior management-Function/Business Unit/Department Heads (4)	54	18.24%	
Top management-Leadership roles, CXOs (5)	40	13.51%	

### Participant’s profile

The socio-demographic profile of the participants is as follows:

## Data analysis

The current study aims to analyse the moderating effect of EI in VCE and DM effectiveness. Hierarchical regression was used to identify the relationship between VCE and DME and the moderating effect of EI on VCE and DME. Since the study used summed scores, usage of this procedure is considered on par with structural equation modelling (SEM) usage (Hayes, 2017). Moreover, PROCESS does not mandate estimation of regression equation parameters since the least square regression program using SPSS will provide similar results as SEM (Singh & Bamel, 2020), and it does not require path analysis as in the case of SEM. To test hypothesis H1: Virtual communication Effectiveness (VCE) influences Decision making effectiveness (DME), a hierarchical regression analysis was conducted. Table 2 depicts the results of the analysis.

**Table 2:** Structural estimates (sample size: 296)

Hypothesis	Effect of	Effect on	β	*p*-value	Results
H1	VCE	DME	1.819	< 0.01	Supported

### Moderation analysis

**Fig. 1:**
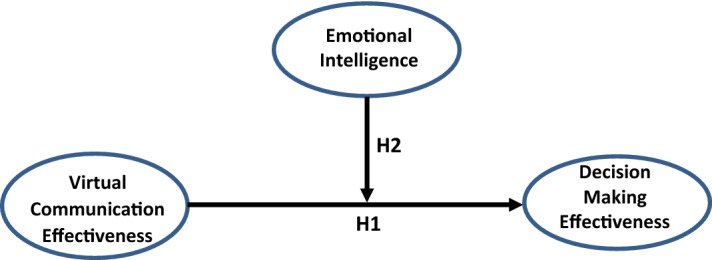
Conceptual framework

The PROCESS Macro version 3.4, model 1 (Hayes, 2017) was used to identify the moderating effect of EI on VCE and DME. In this simple moderation model, the effect of VCE on DME and the interaction effect of VCE and EI are tested (Fig. 1), (Table 3).

**Table 3:** Moderation analysis (sample size: 296)

	Coeff	se	t	*ap*	LLCI	ULCI
Constant	−3.8894	1.4841	−2.6208	.0092	−6.8102	−.9686
EI	1.8221	.3640	5.0055	.0000	1.1057	2.5385
VCE	1.8193	.5552	3.2767	.0012	.7266	2.9120
int_1	−.4657	.1353	−3.4419	.0007	−.7320	−.1994

In this model, it can be seen that conditional effect of VCE on DME is = 1.819 + (-0.4657)* EI. What this means is that the effect of VCE on DME decreases by 0.47 units when the level of EI is increased by one unit. This means that the dependence on technology on VCE can be less when increasing levels of EI. As per the above results, the effect of EI (*p*-value is 0.000) and VCE (*p*-value is 0.0012) on DM have been recorded less than 0.05 which confirms it to be significant at a 5% level of significance (*p* < 0.05). Also, the interaction effect is found significant with the *p*-value of 0.007 which shows that the moderation effect of EI has come significant at a 5% level of significance (*p* < 0.05). Hence, it can be concluded that EI acts as a moderator between VCE and DM. To check the conditions under which the moderation effect has come significant, the conditional effect is checked at three levels shown below such as low, medium, and high. Table 4 shows three levels of moderation. The results show that EI moderates only in two conditions, namely low and high with respective values 0.0369 and 0.0171 (*p* < 0.05). This can be interpreted as follows: When the moderation level is medium, EI does not moderate between VCE and DM but when the level is set as low or high, EI moderates between VCE and DM significantly. This means that when EI levels are low, it moderates the relationship between independent and dependent variables. This means that VC team members will have a greater dependence on technology for improved DME. Similarly, when the EI levels are high, the dependence on technology for the VC team members is relatively less for improved DME.

**Table 4:** Conditional effect of X on Y at values of the moderator (sample size: 296)

EI	Effect	se	t	*p*	LLCI	ULCI
3.3547	.2569	.1225	2.0969	.0369	.0158	.4981
3.8494	.0266	.0815	.3261	.7446	−.01338	.1869
4.3441	−.2038	.0850	−2.3991	.0171	−.3710	−0.366
Data for visualizing the conditional effect of X on Y						
Data list free/CE EI DM						
Begin data						
2.0765	3.3547	2.7568	Low EI			
2.6475	3.3547	2.9035				
3.2185	3.3547	3.0502				
2.7065	3.8494	3.1798	Medium EI			
2.6475	3.8494	3.1949				
3.2185	3.8494	3.2101				
2.0765	4.3441	3.6027	High EI			
2.6475	4.3441	3.4863				
3.2185	4.3441	3.3700				

**Fig. 2:**
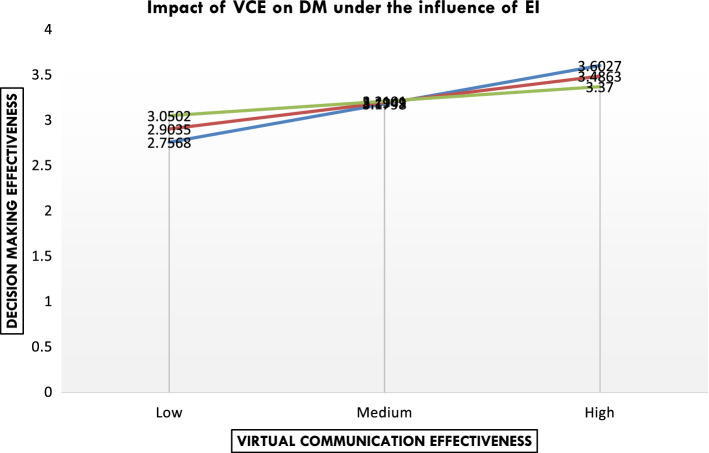
Impact of virtual communication on decision making under the influence of emotional intelligence

Figure 2 shows three conditions of moderation as mentioned below:

- The first condition depicts that when the moderation level is low, high VCE is required to achieve a high level of DM.
- The second condition reveals that when moderation is medium, there is no moderation effect found between VCE and DM.
- The third condition exhibits that when moderation is high, a low level of VCE is required to achieve a high level of DM.

## Discussion

The study examines the association between VC effectiveness and perceived DM effectiveness of the participants and the moderation effect of EI on the association of dependent and independent variables. VCE is found to impact DM effectiveness significantly. This outcome suggests that the participants found that VC can help in improving DM.

EI is found to have a significant moderating effect on the association of VC effectiveness and DME. Since the p-value (*p* ≤ 0.007) is found significant, EI acts as a moderator that affects the strength of the relationship between VC effectiveness and DME. In this study, EI amplifies the relationship between VC and DM. Similar findings by Alward and Phelps (2019) emphasizes the importance of EI in the effectiveness of virtual teams. The positive influence of EI dimensions while addressing the challenges of the virtual project team is reported by Nauman et al (2006), thereby corroborates the current research results. Another study by Mysirlaki and Paraskeva (2020) on a 500 member virtual team reveals that leadership EI has a predictive ability on virtual team effectiveness.

The study also highlights the specific outcomes at different levels of EI of team members. Since the interaction effects are different at different levels of moderation, it brings out a typical influence on the predictor and outcome variables. When the moderator's influence is low, there is a greater need for effective VC for improved DM. It implies that there is a need for greater emphasis on task and technology match, switching the tools and investment in training the team members when the moderation effect of EI is low to enhance DM. When the moderation effect of EI is high, there is less effort that needs to be put on making VC more effective and thereby better DM. Emphasis on the use of technology can be reduced if the team member’s can be trained to improve their EI. Hence, training the VC team members on EI seems to be a long term strategy for DME.

### Theoretical implications

Previous seminal study by Straus and McGrath (1994) compared the F2F and VC in the Task- Technology context and addressed the question “Can medium matter?”. The current study continues the research idea by addressing “How can the medium be made more effective ?”.

This study confirms the TTF theory where there is a definite emphasis on individual characteristics. Manipulation of individual characteristics can have varying effects of task technology fit on the performance of virtual teams. When individual dimensions are augmented (EI in this study), there is greater effectiveness in DM with lesser use of technology in VC. The study confirms the moderation effect under low and high levels of EI. Hence this study provides an original benchmark model for organisations to adopt during the unprecedented times of pandemic.

### Managerial implications

The study suggests EI as a required dimension for improved VCE and DME. The findings are relevant to educators, consultants, and any professional who need to adapt VC platforms on an ongoing basis. For educators, the study findings help in upskilling themselves and be ahead of the learning curve in the context of blended teaching/learning. Inclusion of EI in structuring the teaching, assessment and learning management system (LMS) can effectively enhance the teaching–learning process. Training need analysis (TNA) of the virtual communicators like educators who use the virtual platform more frequently will be supported by this study, where modules of EI training can be a part of any training schedule. A thorough understanding of EI can help the course makers to structure the components of blended learning more effectively. Challenges of VC like co-presence, time zone differences, socio-demographic challenges, cultural challenges, and technostress can be greatly reduced by training the VC team members on EI dimensions.

Higher levels of EI can reduce the dependency on technology leading to desirable financial considerations. Heavy investment in technology and training in new technology can be reduced by one-time training in different dimensions of EI like Self-awareness, Assertiveness, Empathy, Building bonds, Interpersonal relationship, Tolerance to stress, Self-control, Adaptability, and Optimism.

There is a definite need to track the interaction and participation levels of the VC participants. Making use of different software to suit the participants technology comfort level will help in better VC and thereby better DM. The provision of emoticons to communicate emotions while communicating virtually, tend to improve the effectiveness of communication and thereby the effectiveness of DM. Guidelines can be sent to new participants of VC to help them operate in a virtual platform and engage productively with existing members. Providing a climate of openness will allow free flow of communication and thereby multiple alternatives to make informed decisions. An intervention team consisting of Management Information System (MIS) executives, can be exclusively planned for VC set-up to circumvent technology related challenges and help the VC team focus on the task at hand.. Formation and use of an intervention team to ensure regular flow of credible, as well as mandatory information between managers and team members, can be done to avoid an unproductive communication chain.

Effective Stress coping is one of the prominent dimensions of EI. The provision of employee wellness programs helps in effective stress coping. In addition, work-life balance is projected as a constraint in this study and therefore policymakers can consider policy amendments to reduce the stress caused due to VC which intrudes into their personal space.

### Limitations and scope for future research

This research is a pioneer study in the context of studying the moderating effect of EI on VCE and DME. However, the study has a few limitations. Being a cross-sectional study, it has relatively weak internal validity in comparison to experimental research. Since the instrument used is a self-report measure, social desirability-based responses may hamper the accuracy of the responses. Though common method bias and nonresponse bias were corrected while data was made suitable for analysis, this study is not free from the challenges of self-administrative questions. Another limitation of the study is the focus on only individual characteristics of TTF theory. Future studies can include the technology characteristics alongside the individual characteristics to understand the increase in performance to encompass the elements of TTF theory.

Future studies can consider segmenting the participants proportionate to the size of the workforce in each of the industries of study to have better representation and the relative impact of VCE on DME. Replicating the study PAN India can help in organizational policy amendments. An empirical study using the DM style inventory (Scott & Bruce, 1995) can be used to understand the predominant DM style in a VC context and DME. Future studies can consider including social media in the model to test the effectiveness of improved performance as there is an increased thrust on the use of social media. A mixed research method is suggested using qualitative study tools like Focus group discussion and interviews to validate the empirical study findings. Considering the inclusion of personality type as a control variable will add another dimension to the research, as communication effectiveness depends on the personality dimensions.

## Conclusion

The study explores the moderation effect of EI in VC context. Communication in the virtual environment necessitates adopting newer ways of people engagement, and the stakeholders need to take cognizance of the fact that human factors are more important than technological factors in the unprecedented new normal. This paper is an amalgamation of empirical study and relevant literature reviews to connect critical decision-making in a virtual environment. The study confirms that EI moderates the relationship between VCE and DME. By augmenting one of the individual characteristics (EI), there can be less reliance on the technology used to meet efficiency in decision-making in the VC context. The necessity to communicate seamlessly is the order of the day, and technology-dependent communication is the way forward. Early adoption of technology by the stakeholders can go a long way in future readiness. This research concludes that organizations can achieve their objectives successfully by strengthening the VC infrastructure and providing a non-threatening platform of communication for the team members.

Therefore, an optimal blend of task technology and individual characteristics will greatly improve the performance of the VC team and hence lead to effective DM. The study thus underlines the role of EI in VC mode for DME.
